# Effects of Mix Components on Fracture Properties of Seawater Volcanic Scoria Aggregate Concrete

**DOI:** 10.3390/ma17164100

**Published:** 2024-08-19

**Authors:** Yijie Huang, Lina Zheng, Peng Li, Qing Wang, Yukun Zhang

**Affiliations:** 1 College of Civil Engineering and Architecture, Shandong University of Science and Technology, Qingdao 266590, China; 13583813525@163.com (L.Z.); lipeng@sdust.edu.cn (P.L.); philc007@163.com (Y.Z.); 2 College of Mechanical and Architectural Engineering, Taishan University, Tai’an 271000, China; qwang@sdust.edu.cn

**Keywords:** seawater sea sand volcanic scoria concrete, fracture properties, double-*K* fracture parameters, softening curve, constitutive model, digital image correlation

## Abstract

The fracture mechanism and macro-properties of SVSAC were studied using a novel test system combined with numerical simulations, which included three-point bending beam tests, the digital image correlation (DIC) technique, scanning electron microscopy (SEM), and ABAQUS analyses. In total, 9 groups and 36 specimens were fabricated by considering two critical parameters: initial notch-to-depth ratios (*a*_0_/*h*) and concrete mix components (seawater and volcanic scoria coarse aggregate (VSCA)). Changes in fracture parameters, such as the load-crack mouth opening displacement curve (*P*-CMOD), load-crack tip opening displacement curve (*P*-CTOD), and fracture energy (*G*_f_), were obtained. The typical double-*K* fracture parameters (i.e., initial fracture toughness ($K_{\mathrm{IC}}^{\mathrm{ini}}$) and unstable fracture toughness ($K_{\mathrm{IC}}^{\mathrm{un}}$)) and tension-softening (*σ*-CTOD) curve were analyzed. The test results showed that the initial cracking load (*P*_ini_), *G*_f_, and characteristic length (*L*_ch_) of the SVSAC increased with decreasing *a*_0_/*h*. Compared with the ordinary concrete (OC) specimen, the *P*-CMOD and *P*-CTOD curves of the specimen changed after using seawater and VSCA. The evolution of the crack propagation length was obtained through the DIC technique, indicating cracks appeared earlier and the fracture properties of specimen decreased after using VSCA. Generally, the $K_{\mathrm{IC}}^{\mathrm{un}}$ and $K_{\mathrm{IC}}^{\mathrm{ini}}$ of SVSAC were 36.17% and 8.55% lower than those of the OC specimen, respectively, whereas the effects of *a*_0_/*h* were negligible. The reductions in *P*_ini_, *G*_f_, and *L*_ch_ of the specimen using VSCA were 10.94%, 32.66%, and 60.39%, respectively; however, seawater efficiently decreased the negative effect of VSCA on the fracture before the cracking width approached 0.1 mm. Furthermore, the effects of specimen characteristics on the fracture mechanism were also studied through numerical simulations, indicating the size of the beam changed the fracture toughness. Finally, theoretical models of the double-*K* fracture toughness and the *σ*-CTOD relations were proposed, which could prompt their application in marine structures.

## 1. Introduction

The construction of marine islands and reefs has become an urgent issue owing to the implementation of a strategy for building a powerful marine country, particularly for China’s large economic need [1]. Seawater volcanic scoria aggregate concrete (SVSAC) is an advantageous option for marine construction owing to its advantages such as locally available material and huge deposits, which can shorten the construction period and reduce the cost. For SVSAC, the coarse aggregates, mixed water, and fine aggregates are volcanic scoria coarse aggregate (VSCA), seawater (SW), and sea sand (SS), respectively [2]. Volcanic scoria aggregates (VSAs) are characterized by a porous structure, low elastic modulus, porosity, and light weight [3,4]. SS and SW contain numerous chloride ions (Cl^−^) and sulphates (SO_4_^2−^). Thus, the properties of SVSAC are more complex than those of ordinary concrete (OC) and require further investigation. 

The mix components of SVSAC (VSCA, SW, and SS) are the main causes of their complex behaviors. In general, VSCA is obtained from volcanic scoria which is an important kind of pyroclastic material and has the basic composition of basalt [5]. The volcanic scoria aggregates have been extensively adopted in past construction [6,7,8,9]. The ancient Roman architects started to adopt volcanic scoria aggregates to fabricate mortars or ‘Roman concrete’ with special characteristics from the first century before Christ (e.g., Pantheon (Rome), the monuments of imperial age in Rome) [10,11]. The influence of VSCA on concrete properties (e.g., strength, ductility, and fire resistance) has been studied through several analyses [12,13,14]. The test results indicated that the lightweight and porous structure of VSCA decreased the concrete density, resulting in a lower deformability compared to that of the OC by approximately 15–30%. Hossian [15] revealed that VSCA had a detrimental influence on the workability of concrete, and the slump of concrete was acceptable by preparing VSCA under saturated-surface dry conditions [16]. VSCA can also be used to develop a series of concrete grades that exhibit sufficient desirable strength and acceptable durability in the structural range. The results indicated that after substituting VSCA for gravel, the concrete compressive and tensile strengths decreased by approximately by 8.35–40% and 2–30%, respectively [17,18], and the ratio of tensile strength to compressive strength of concrete with VSCA was 0.08–0.11. The increase in concrete strength can reach 50% when river sand (RS) is replaced with volcanic scoria fine aggregate (VSFA), owing to the desirable particle size distribution and granular properties of VSFA [19]. The elastic and rupture modulus of specimen adopting VSCA were 10–21 GPa and 2.7–4.8 MPa, respectively [20], and the variation in the concrete’s Poisson’s ratio (0.19–0.21) was negligible. In general, the stress–strain relationship of concrete adopting VSCA under uniaxial compression is different from that of OC [21,22]. SW and SS also significantly affected the properties of the concrete. The addition of SS improved the slump and fluidity of concrete, whereas a high shell content significantly affected the workability of the concrete. The carbonation, Cl^−^ penetration, and freezing resistances of concrete adopting SW and SS were improved compared to those of OC [23,24,25]. For the underwater structures using Roman concrete, SW caused an immediate chemical reaction, and hydrated silicates and calcium aluminosilicates were formed and C-S-H phases were created, which ensured the connection of all ingredients [7]. Xiao [26], Huang et al. [27], and Sariman et al. [28] studied the compressive strength of seawater sea sand concrete at different ages and revealed that its strength developed rapidly during the early ages owing to the high Cl^−^ content, which accelerated the hydration reaction of cement. However, the development of strength was delayed after 28 days, and the ductility of concrete with SW and SS decreased slightly [29]. Huang et al. [2] investigated the mechanical properties of concrete by simultaneously adopting the VSCA, SW, and SS under axial compression. It was observed that the specimen failed suddenly, and its deformability and elastic modulus were approximately 20.5% and 31.7% inferior to those of OC, respectively. Based on the above findings, the workability, strength, and durability of SVSAC met the construction requirements. However, the cracking of SVSAC was different from that of OC, which reduced the deformability and ductility of the specimen. 

Fracture investigations can reveal the principle of material cracking (initiation and propagation of cracks), which can improve the behavior of structures. Several research works [30,31] obtained the evolution of the crack propagation length based on digital image correlation (DIC) technology and verified the reliability of DIC. Gui et al. [32] systematically studied the fracture properties of concrete containing SW and SS. The fracture toughness of the specimens increased with the maximum aggregate size. Zang et al. [33] and Zhou et al. [34] reported that concrete mixed with SW and SS, replacing freshwater and RS, can improve the unstable fracture toughness and fracture energy and decrease its brittleness. Zhou et al. [35] proposed that coral concrete is more brittle, less ductile, and prone to cracking, whereas seawater immersion curing can improve its hydration degree of coral concrete and densify the interfacial transition zone (ITZ) between the coral coarse aggregate and cement matrix. However, to the best of our knowledge, few studies have focused on the fracture properties of concrete using VSCA, and the coupled effects of SW, SS, and VSCA on the fracture of concrete have not yet been studied. Further tests and theoretical analyses are required to confirm this hypothesis. Thus, a systematic investigation on the fracture properties of SVSAC was undertaken in this study through a novel test system combined with numerical simulations, which included three-point bending beam tests, the digital image correlation (DIC) technique, scanning electron microscopy (SEM), and ABAQUS analyses. The macro fracture performance and mechanism of SVSAC are investigated by this test system.

## 2. Materials and Methods

### 2.1. Raw Materials

The main parameters in this study were the initial notch-to-depth ratios (*a*_0_/*h*: 0.2, 0.3, and 0.4) and the concrete mix components (SW, SS, and VSCA). Considering the effects of the mix components, three types of concrete were used in the test, i.e., OC, SVSAC, and volcanic scoria aggregate concrete (VSAC). Notably, the mixed water and fine and coarse aggregates of the VSAC were fresh water (FW), RS, and VSCA, respectively. 

**Figure 1:**
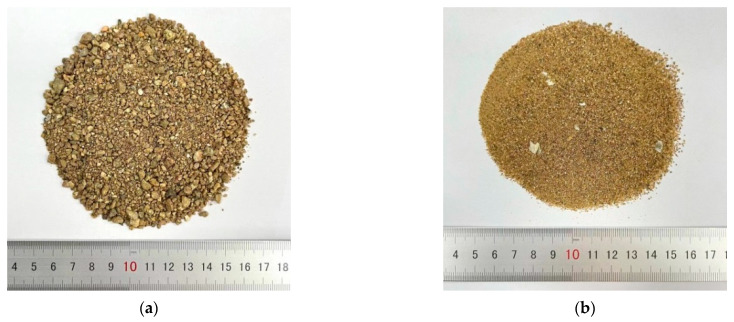
Fine aggregates: (**a**) RS; (**b**) SS.

**Figure 2:**
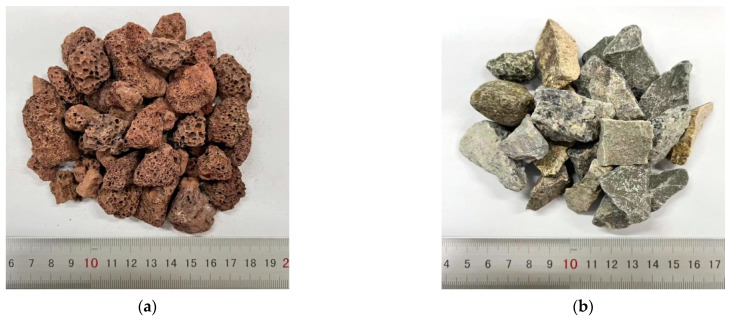
Coarse aggregates: (**a**) VSAC; (**b**) Gravel.

The mixing water in the test included SW and freshwater, and the fine aggregates consisted of SS and RS (Figure 1). To determine the effects of coarse aggregates on fracture performance, two types of coarse aggregates were considered: VSCA and gravel (Figure 2). P.O 42.5 ordinary cement and an HSC polycarboxylic acid high-performance water-reducing agent were used to fabricate the specimens. The basic properties and compositions of the aggregates are listed in Table 1.

**Figure 3:**
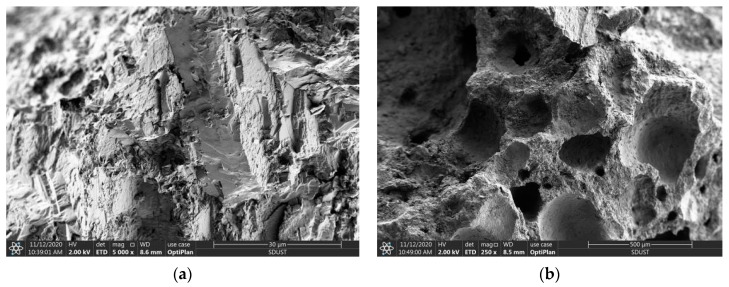
SEM of aggregates: (**a**) Gravel; (**b**) VSCA.

Compared with gravel (Table 1), the *ω*_a_ and CR of VSCA increased by 1134.17% and 210.95%, respectively; however, its *ρ*_b_ and *ρ*_a_ were 54.62% and 36.41% lower, respectively. The lightweight and porous structure of volcanic scoria changed the properties of VSCA (Figure 3). SS had a higher *α*, while its CL was acceptable. SW was obtained from Lingshan Bay, Qingdao, China. The main chemical compositions of SW were Cl^−^ (19.83 g/L), Na^+^ (9.22 g/L), SO_4_^2−^ (2.31 g/L), Mg^2+^ (1.12 g/L), and Ca^2+^ (0.34 g/L), etc.

### 2.2. Concrete Mix

The target concrete strength adopted was C30 because of its wide application in practical engineering [36,37]. Three types of concrete were prepared: OC, VSAC, and SVSAC, to systematically study the effects of the concrete mix components on the fracture properties of the SVSAC specimen. The details of the concrete mix were determined through preliminary tests, as listed in Table 2. The mix proportions of VSAC and SVSAC were different from those of OC owing to the properties of the aggregates. 

### 2.3. Experiment Design

#### 2.3.1. Specimen Design and Fabrication

The effects of the concrete mix components (SW, SS, and VSCA) and initial notch-to-depth ratios (*a*_0_/*h* = 0.2, 0.3, and 0.4) on the fracture properties of SVSAC were investigated using 9 groups and 36 three-point bending beams. According to RILEM50-FMC [38] and the related code, the specific size of the beam was maintained at 200 × 100 × 1190 mm^3^ (width × height × length), and the initial crack width was 3 mm. 

The specimens were named according to the following rules (Table 3): concrete type (OC, VSAC, and SVSAC), *a*_0_/*h* (0.2, 0.3, and 0.4). Taking SVSAC-0.2 as an example, “SVSAC” and “0.2” denote seawater volcanic scoria aggregate concrete and a 0.2 initial notch-to-depth ratio, respectively.

**Figure 4:**
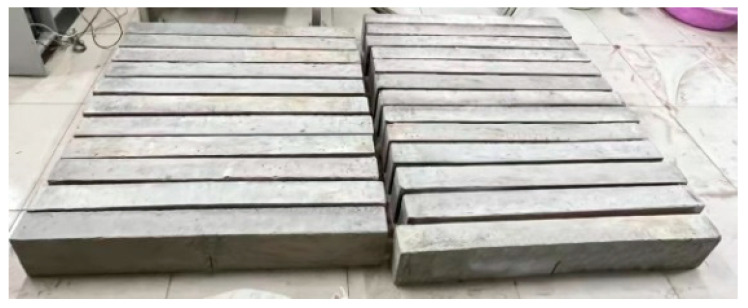
Typical specimens.

The specimens were fabricated as follows. First, the concrete mixture was filled into a test mold and vibrated uniformly. Second, prefabricated cracks were obtained by carefully removing the prebuilt steel plates (3 mm thickness) after the initial setting of the concrete (3 h). Third, the mold was removed after 24 h, and the specimens were cured under standard conditions for 28 d (temperature: 23 ± 2 °C, humidity: 95 ± 3%). The actual beam specimens are shown in Figure 4.

#### 2.3.2. Loading Setup and Program

**Figure 5:**
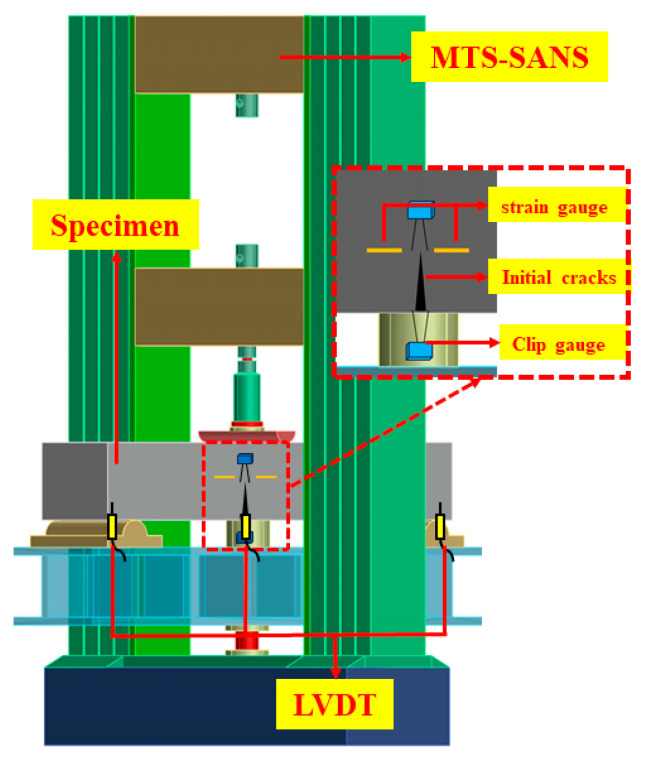
Specimen loading diagram.

The loading setup for the fracture studies included three parts: an MTS-SANS (MTS Corporation, Eden Prairie, MN, USA) (30 kN) electronic universal testing system, a measuring system (i.e., clip gauges, strain gauges, and linear variable differential transformers (LVDTs)), and a computer system, as shown in Figure 5. Clip gauges with a maximum range of 50 mm were placed at the bottom and tip ends of the notch to measure the crack mouth opening displacement (CMOD) and crack tip opening displacement (CTOD) during the test. The LVDTs (50 mm) were arranged at the midspan and ends of the beam to obtain the deflection. Furthermore, four strain gauges were symmetrically attached to the pre-notched tip on both sides of the beam to determine the crack initiation (Figure 5). 

**Figure 6:**
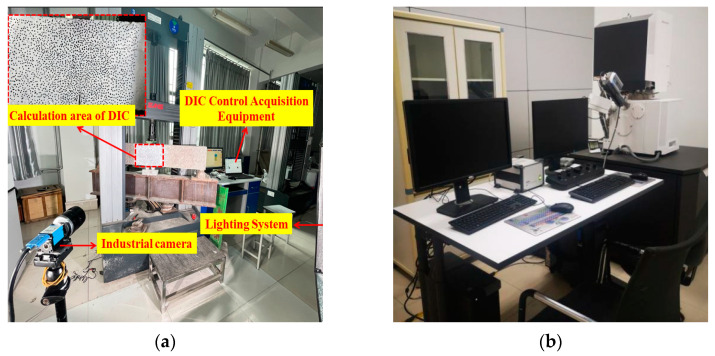
Experimental equipment: (**a**) Illustration of actual loading system DIC test; (**b**) Apreo S HiVac SEM system.

The digital image correlation (DIC) technique was adapted to measure the full-field surface displacement of the beam and obtain the crack propagation and characteristics such as the crack opening displacement (COD) and crack propagation length (*l*_l_). An area of 200 × 100 mm^2^ above the notch was prepared using the DIC technique (Figure 6a). Images of the specimen during the test were captured and analyzed using the DIC technique (aperture value of *f*/6.3, exposure time of 1/13 s, and image acquisition frequency of 0.5 Hz). It should be noted that the specimen was positioned perpendicularly to the industrial camera to reduce errors.

In addition, a vacuum control system, a signal detection and image display system, and an electronic optical system were utilized in conjunction, collectively comprising an Apreo S HiVac scanning electron microscope (Thermo Fisher Scientific, Shanghai, China) (SEM) system (Figure 6b) to analyze the microstructure of concrete and SVSAC. The dimensions of the specimens were consistently maintained at 5 mm × 5 mm × 2 mm.

Displacement control was used during the test, and the loading rate was maintained at 0.02 mm/min with a data acquisition frequency of 30 Hz. A 1 kN load was applied to the beam to ensure normal operation of the loading system before the actual test. Furthermore, digital images of the deformed specimens were captured using an industrial camera every 2 s to record the related information.

## 3. Results

### 3.1. Failure Phenomenon

#### 3.1.1. Failure Pattern

**Figure 7:**
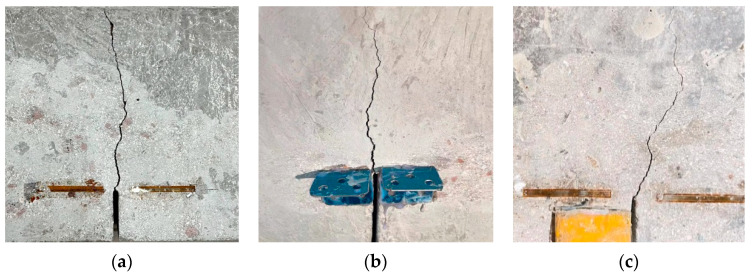
Concrete crack propagation: (**a**) VSAC-0.3; (**b**) SVSAC-0.3; (**c**) OC-0.3.

The failure of the SVSAC three-point bending beam was similar to that of the OC and VSAC specimens. The initial cracking of the beam first appeared at the tip of the notch when the vertical load (*P*) reached approximately 60–70% of peak load (*P*_max_). The number of cracks increased with increasing *P*. The cracks propagated approximately parallel to the loading direction (Figure 7). Deformation and cracks in the beam became evident when *P* approached *P*_max_. After the peak point, *P* decreased rapidly, and cracks crossed the entire section of the beam. The specimens failed suddenly. 

**Figure 8:**
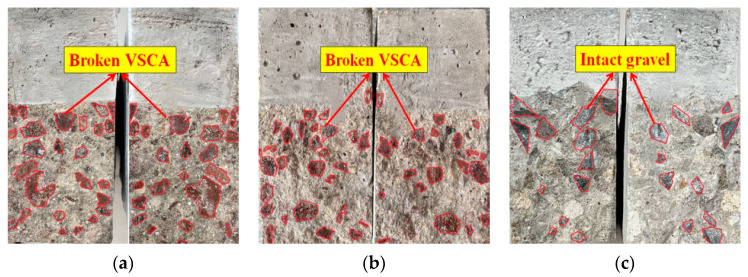
The typical fracture surfaces of beams: (**a**) VSAC-0.4; (**b**) SVSAC-0.4; (**c**) OC-0.4.

The typical fracture surfaces of the beams are shown in Figure 8. Compared to OC, the aggregates of VSAC and SVSAC were almost damaged, and their fracture surfaces were smooth, irrespective of *a*_0_/*h* (Figure 8). This can be attributed to the cracks that can easily cross the porous and lightweight VSCA. SS and SW had negligible effects on the fracture surface, and the failure pattern of VSAC was similar to that of SVSAC.

#### 3.1.2. Crack Propagation and Characteristics

**Figure 9:**
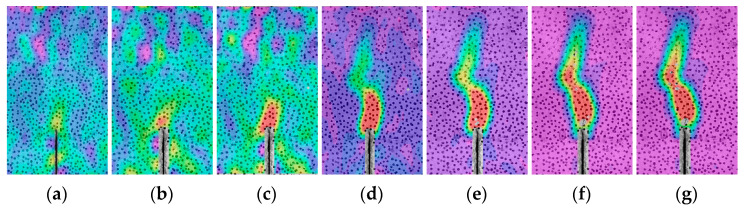
Strain distribution evolution of OC-0.3 during the loading period: (**a**) Pre-80%; (**b**) *P*_max_; (**c**) Post-80%; (**d**) Post-60%; (**e**) Post-50%; (**f**) Post-30%; (**g**) Post-15%.

**Figure 10:**
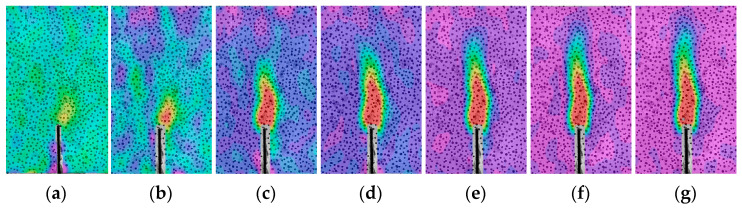
Strain distribution evolution of SVSAC-0.3 during the loading period: (**a**) Pre-80%; (**b**) *P*_max_; (**c**) Post-80%; (**d**) Post-60%; (**e**) Post-50%; (**f**) Post-30%; (**g**) Post-15%.

**Figure 11:**
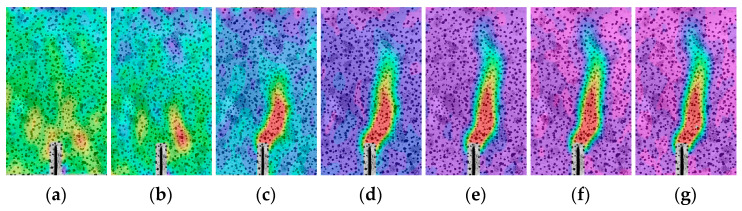
Strain distribution evolution of VSAC-0.3 during the loading period: (**a**) Pre-80%; (**b**) *P*_max_; (**c**) Post-80%; (**d**) Post-60%; (**e**) Post-50%; (**f**) Post-30%; (**g**) Post-15%.

**Figure 12:**
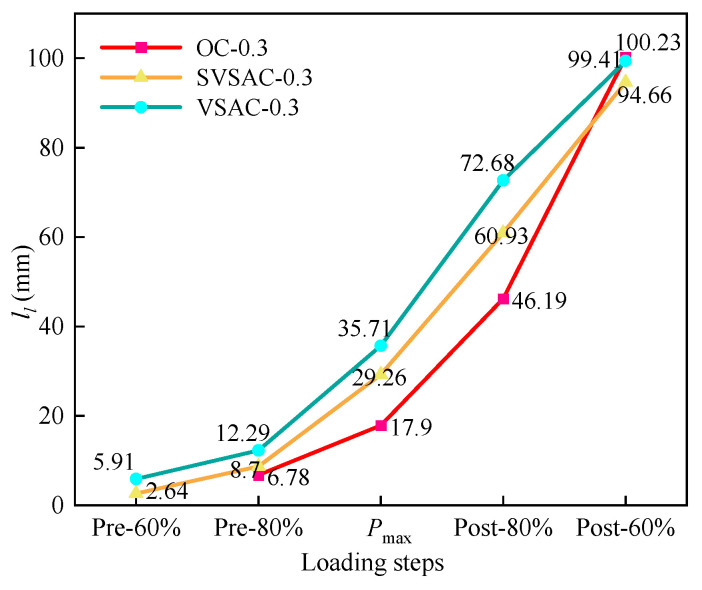
The crack propagation length during different loading steps.

**Figure 13:**
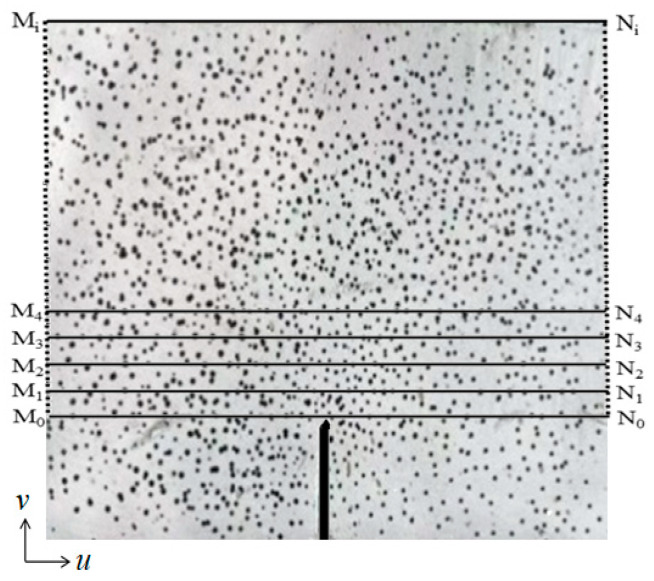
Calculation lines of DIC.

**Figure 14:**
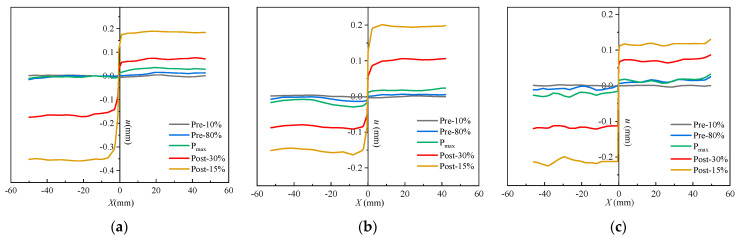
COD at M_0_N_0_ during different loading periods: (**a**) OC-0.3-4; (**b**) SVSAC-0.3-4; (**c**) VSAC-0.3-4.

The crack propagation during the fracture process of the concrete specimens in this study was recorded using the DIC technique (Figure 9, Figure 10 and Figure 11). The displacement jump map obtained by DIC was also adopted to reveal the development of crack characteristics (COD and *l*_l_) (Figure 12, Figure 13 and Figure 14). The calculation method is described in Refs. [39,40]. The crack characteristics were negligible during the early loading stage.

1. 1. Crack propagation pattern;

The strain distributions of the specimens during the tests are shown in Figure 9, Figure 10 and Figure 11. The initial cracking of the beam appeared on the tip of the notch when the vertical load (*P*) reached approximately 80% of the peak load (*P*_max_), and the fracture process zone (FPZ) of the specimen gradually increased as *P* increased, particularly when *P* decreased to approximately 80% of *P*_max_. When *P* decreased to 60% of *P*_max_, the FPZ expanded rapidly, and permanent cracks were formed which is in accordance with the observed failure pattern of the specimen (Section 3.1.1).

2. Crack characteristics.

DIC accurately captured the development of *l*_l_ and COD during the fracture failure process (Figure 12). It can be observed that *l*_l_ evidently changes with variations in the concrete mix components. When *P* reached 60% of *P*_max_, the *l*_l_ values of SVSAC-0.3 and VSAC-0.3 were 2.64 mm and 5.91 mm, respectively, while the *l*_l_ of OC-0.3 was negligible. The cracks in the specimen appeared earlier after adopting volcanic scoria, owing to the lightweight and porous structure of VSCA.

**Figure 15:**
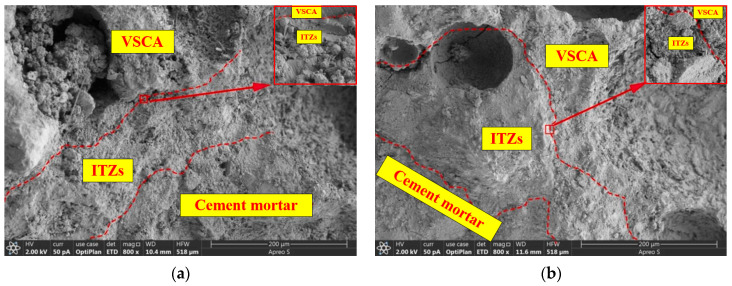
Microstructures of VSAC and SVSAC: (**a**) VSAC; (**b**) SVSAC.

When *P* approached *P*_max_, the CTOD values (COD at M_0_N_0_, Figure 13) of OC-0.3, SVSAC-0.3, and VSAC-0.3 were 0.041, 0.043, and 0.044 mm, respectively. However, the CTOD of OC-0.3 was 19.17% and 19.66% greater than those of VSAC-0.3 and SVSAC-0.3 when *P* decreased to 30% of *P*_max_, respectively (Figure 14). In addition, the *l*_l_ value of SVSAC-0.3 was 28.32% greater than that of OC-0.3, whereas the *l*_l_ of VSAC-0.3 increased by 41.26% compared with that of SVSAC-0.3, revealing that the negative effect of VSCA on the cracking of concrete was reduced by SW and SS. This is because the Cl^−^ in the SS and SW reacts with cement and yields Friedel’s salt, which fills the internal pores and improves the microstructure of the concrete (Figure 15). After the peak point, cracking of the specimen developed rapidly, and the difference between the *l*_l_ value of SVSAC-0.3 and that of VSAC-0.3 became minor (5.01%) when *P* decreased to 60% of *P*_max_. The influences of SS and SW on the *l*_l_ value of SVSAC decreased at the final stage of loading owing to the failure of the microstructure of the concrete.

### 3.2. Physical Properties of SVSAC

The physical properties of concrete were obtained based on standard codes [41,42]. The cubic compressive strength (*f*_cu_) of OC and VSAC was 38.3 MPa and 37.5 MPa, respectively (Table 4), which meets the requirements of this study. However, the splitting tensile and prismatic compressive strengths (*f*_c_ and *f*_t_) of SVSAC increased by 12.04% and 17.31%, respectively, compared to those of OC, even under a similar *f*_cu_. The high mechanical interlock between VSCA and cement paste could inhibit the development of microcracks and cause a higher *f*_t_. Compared to the elastic modulus (*E*_c_) and apparent density (*ρ*_d_) values of OC, those of SVSAC decreased by 29.34% and 16.85%, respectively, owing to the lightweight and porous structure of scoria aggregates.

### 3.3. Fracture Characteristic Loads

#### 3.3.1. *P*_ini_

The crack initiation load (*P*_ini_) and *P*_max_ of the SVSAC were determined using the strain gauge method [43]. Compared to the *P*_ini_ of OC, that of the specimen adopting VSCA was reduced by 10.94% on average (Table 4). However, SW and SS can decrease the negative effects of the VSCA on the initial cracking of concrete. The results indicated that the *P*_ini_ of SVSAC specimens was 4.98% higher than that of VSAC. The specific reasons are as follows: first, the porous structure and inferior properties of VSCA (i.e., modulus and strength) could not effectively inhibit the initiation of cracks, causing a lower *P*_ini_; second, the high Cl^−^ content promoted the hydration of cement and obtained more Friedel’s salts, resulting in the dense microstructure of concrete and improvements in the *P*_ini_ [44]. 

**Figure 16:**
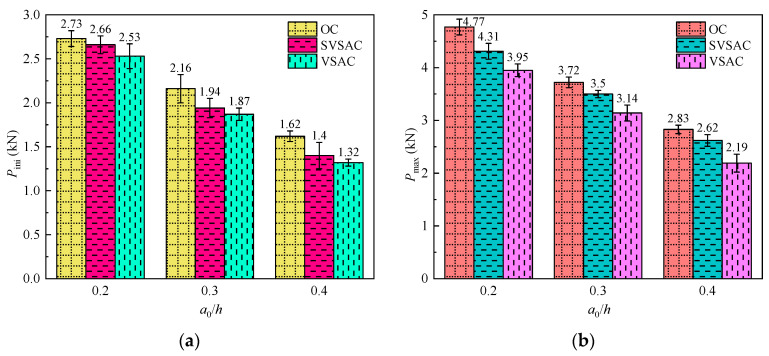
The variation in fracture characteristic loads: (**a**) *P*_ini_; (**b**) *P*_max_.

The *P*_ini_ of SVSAC generally decreased with increasing notch-to-depth ratio (Figure 16a). It was obtained that the *P*_ini_ of SVSAC-0.2 was 36% and 90% higher than those of SVSAC-0.3 and SVSAC-0.4, respectively. The SW, SS, and VSCA changed the influence of *a*_0_/*h* on *P*_ini_. Compared to SVSAC, the *P*_ini_ of VSAC-0.2 was 36.29% and 91.67% greater than those of VSAC-0.3 and VSAC-0.4, respectively, and the effect of *a*_0_/*h* on *P*_ini_ was reduced. 

#### 3.3.2. *P*_max_

The *P*_max_ of SVSAC decreases with increasing *a*_0_/*h* (Table 4, Figure 16b). The *P*_max_ of SVSAC-0.2 was 23.14% and 64.50% higher than those of SVSAC-0.3 and SVSAC-0.4, respectively. Furthermore, *P*_max_ changed with variations in the concrete mix components. It was found that the *P*_max_ of the VSCA specimen decreased by 9.47% on average compared with that of OC; however, the Cl^−^ in SW and SS can hydrate with cement and improve the microstructure of concrete, causing the *P*_max_ of SVSAC to be 13.47% greater than that of VSAC.

### 3.4. P-CMOD and P-CTOD Curves

**Figure 17:**
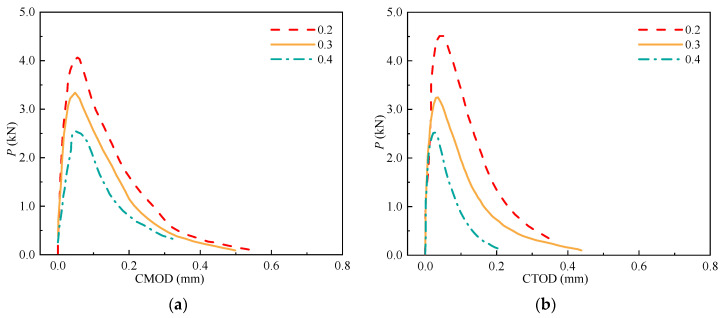
*P*-CMOD and *P*-CTOD of the SVSAC specimen: (**a**) *P*-CMOD; (**b**) *P*-CTOD.

**Figure 18:**
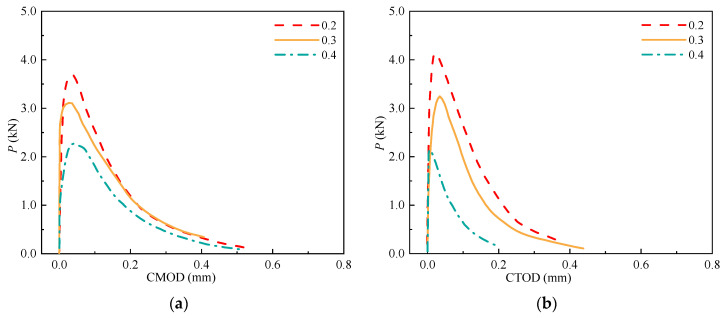
*P*-CMOD and *P*-CTOD of the VSAC specimen: (**a**) *P*-CMOD; (**b**) *P*-CTOD.

**Figure 19:**
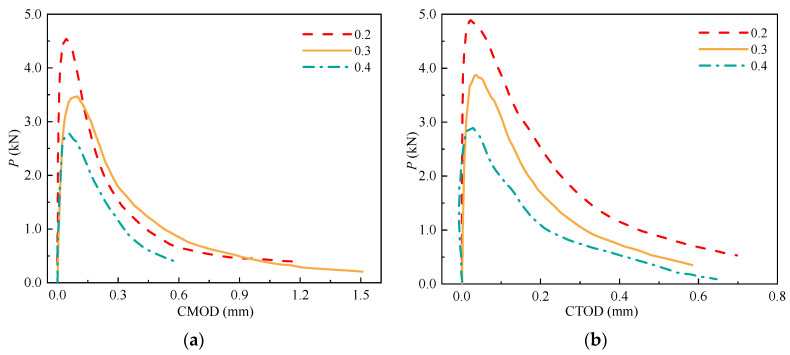
*P*-CMOD and *P*-CTOD of the OC specimen: (**a**) *P*-CMOD; (**b**) *P*-CTOD.

The *P*-CMOD and *P*-CTOD curves are critical for determining the fracture properties of concrete (e.g., *P*_ini_, *P*_max_, and *σ*-CTOD relations). The *P*-CMOD and *P*-CTOD curves of the SVSAC specimens are shown in Figure 17 and can be divided into the initial linear ascending, crack propagation, and fracture failure sections. No visible cracks appeared in the SVSAC-notched beams when the curves were initially in the linearly ascending stage. Microcracks appeared at the notch tips, and the relationship between *P* and CMOD (CTOD) varied when *P* reached approximately *P*_ini_. In general, *P* increases nonlinearly with increasing CMOD (CTOD) during crack propagation. After *P*_max_, *P* declined dramatically and the CMOD (CTOD) continued to increase, indicating that the curves were in the fracture failure stage. Generally, the shape of the curve of SVSAC was similar to that of the curve of OC; however, several differences could be obtained. The mix components changed the development of *P*-CMOD and *P*-CTOD in concrete. Compared with OC, the decline in the *P* of the notched beams adopting VSCA became more rapid after the peak point, indicating the brittleness of SVSAC and VSAC (Figure 17, Figure 18 and Figure 19).

### 3.5. Fracture Energy and Characteristic Length

#### 3.5.1. Fracture Energy

**Figure 20:**
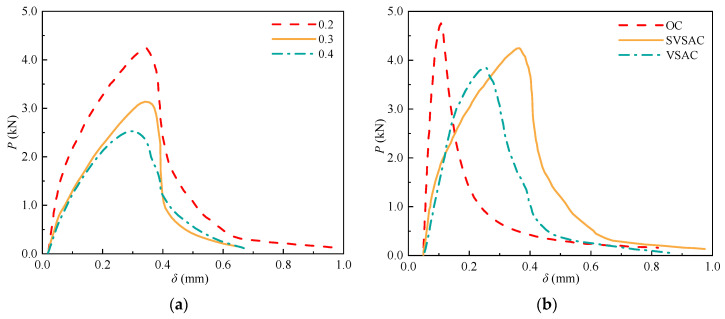
*P*-*δ* curve: (**a**) *P*-*δ* curve of SVSAC under different *a_0_*/*h*; (**b**) Effect of mix components on the *P*-*δ* curve.

*P*-*δ* curves were adopted to calculate the energy dissipation during the fracture process, and the details for the *P*-*δ* curves of concrete beams under different *a*_0_/*h* and mix components are shown in Figure 20a,b.

The energy required for the initiation of concrete cracks per unit area is denoted as the fracture energy (*G*_f_), which reflects the energy dissipation during crack propagation. According to RILEM50-FMC [45,46] and Hillerborg et al. [47], the *G*_f_ of SVSAC can be obtained by *P*-*δ* and Equation (1).

(1) $$G_{f}=\frac{W_{0}+2P_{w}\delta_{0}}{\left(h-a_{0}\right)t}$$

*W*_0_ represents the enclosed area below the measured *P*-CMOD curve, *P*_w_ is the self-weight of the beam, *δ*_0_ is the value of *δ* at *P* = 0, and *t* and *h* are the width and height of the concrete specimens, respectively.

**Figure 21:**
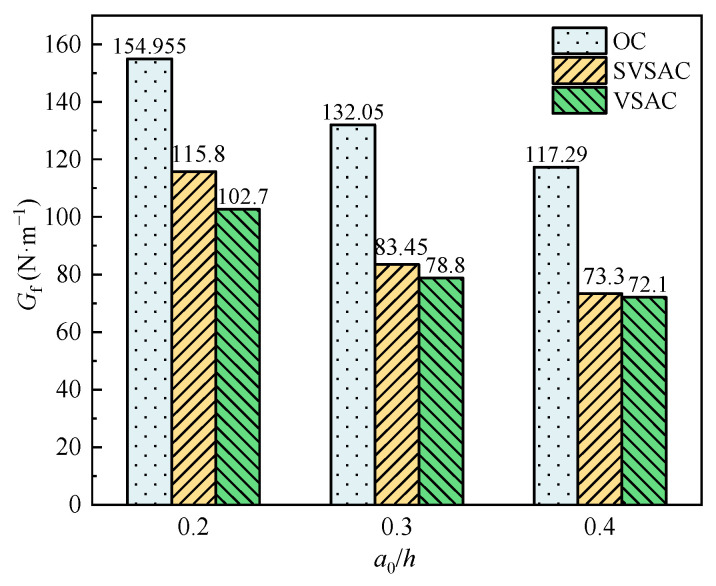
*G*_f_ with different *a*_0_/*h*.

**Figure 22:**
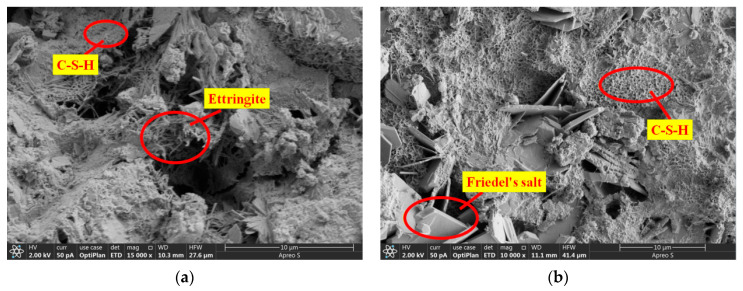
Changes in concrete microstructure after using SW and SS: (**a**) OC; (**b**) SVSAC.

The results revealed that the *G*_f_ of concrete with VSCA was inferior to that of OC under the same concrete strength grade (Figure 21). Compared to OC, the *G*_f_ of SVSAC and VSAC decreased by 32.59% and 37.27%, respectively. The energy dissipation capacity of concrete was significantly reduced after using VSCA, owing to the brittle nature of VSCA and low crack openings, which require less energy for crack propagation. However, SW and SS improved the *G*_f_ of concrete. The fracture energy of SVSAC increased by 7.47% compared with that of VSAC. The higher the Cl^−^ content, the denser the concrete microstructure (Figure 22), the lower the crack propagation, and the greater the *G*_f_ of the concrete.

*G*_f_ decreases with increasing *a*_0_/*h*. The *G*_f_ of OC-0.2 was 17.35% and 32.11% higher than those of OC-0.3 and OC-0.4, respectively. Furthermore, VSCA enhanced the effect of *a*_0_/*h* on *G*_f_, and the *G*_f_ of SVSAC-0.2 was 27.9% and 36.7% higher than those of SVSAC-0.3 and SVSAC-0.4, respectively.

#### 3.5.2. Characteristic Length

The characteristic length (*L*_ch_) proposed by Hillerborg et al. [47] was adopted to reflect the brittleness of concrete, as expressed in Equation (2).

(2) $$L_{ch}=\frac{E_{c}G_{f}}{f_{t}^{2}}$$

*E*_c_ and *f*_t_ denote the elastic modulus and splitting tensile strength of concrete, respectively.

**Figure 23:**
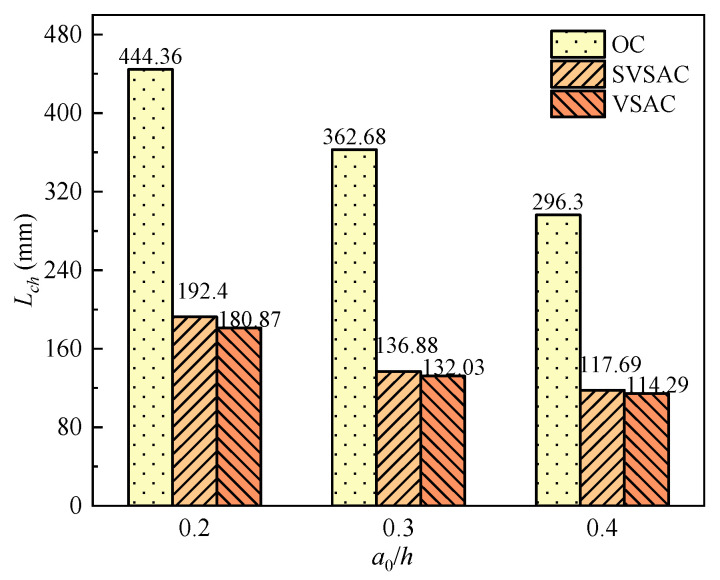
*L*_ch_ with different *a*_0_/*h*.

The effects of *a*_0_/*h* and the concrete mix components on *L*_ch_ (Figure 23) were similar to those of *a*_0_/*h* and the mix components on *G*_f_. Generally, the *L*_ch_ values of SVSAC and VSAC were, on average, 59.49% and 61.28% lower than those of OC, respectively, which indicated the brittleness of concrete containing VSCA. The *L*_ch_ of the VSAC specimens was improved using SW and SS. It was found that the microstructure of the concrete was enhanced because the ions in the SS and SR were involved in the hydration reaction of the cement and the formation of salts, such as Friedel’s filling of the voids in the VSCA. 

The *L*_ch_ of SVSAC decreased and exhibited greater brittleness as *a*_0_/*h* increased. The results indicated that the *L*_ch_ of SVSAC-0.2 increased by 40.56% and 63.48% compared to those of SVSAC-0.3 and SVSAC-0.4, respectively, revealing the rapid propagation of cracks and the high brittleness of concrete with a high initial crack length.

### 3.6. Double-K Fracture Parameters and Tension-Softening (σ-CTOD) Relations

Based on the above analyses, the fracture performance of SVSAC changed with variations in the concrete mix components and *a*_0_/*h*. Thus, the typical fracture properties such as crack initiation and instability were evaluated by using the double-*K* fracture toughness and tension-softening relations (*σ*-CTOD) in detail. 

#### 3.6.1. Double-*K* Fracture Toughness

1. Determination of fracture toughness;

$K_{\mathrm{IC}}^{\mathrm{ini}}$ and $K_{\mathrm{IC}}^{\mathrm{un}}$ have always been employed to describe the crack initiation and instability of concrete based on Xu et al. [48,49]. $K_{\mathrm{IC}}^{\mathrm{ini}}$ represents the ability of concrete to inhibit cracks, and $K_{\mathrm{IC}}^{\mathrm{un}}$ denotes the capacity of concrete to maintain the stable propagation of cracks. Generally, cracks could not develop when the stress intensity factor (*K*) was less than $K_{\mathrm{IC}}^{\mathrm{ini}}$ (*K* < $K_{\mathrm{IC}}^{\mathrm{ini}}$); cracks propagated stably when $K_{\mathrm{IC}}^{\mathrm{ini}}$ < *K* < $K_{\mathrm{IC}}^{\mathrm{un}}$; and cracks developed dramatically when *K* > $K_{\mathrm{IC}}^{\mathrm{un}}$. 

$K_{\mathrm{IC}}^{\mathrm{ini}}$ and $K_{\mathrm{IC}}^{\mathrm{un}}$ are expressed as (Equations (3) and (4)):

(3) $$K_{IC}^{ini}=\frac{1.5\left(P_{ini}+\frac{mg}{2}\times 10^{-2}\right)\times 10^{-3}\times S\times a_{0}^{\frac{1}{2}}}{th^{2}}f\left(\alpha_{0}\right),$$

(4) $$K_{IC}^{un}=\frac{1.5\left(P_{\max}+\frac{mg}{2}\times 10^{-2}\right)\times 10^{-3}\times S\times a_{c}^{\frac{1}{2}}}{th^{2}}f\left(\alpha_{c}\right),$$

where *t*, *h*, *m,* and *S* represent the thickness, height, self-weight, and span of the beam, respectively, *g* = 9.8 m/s^2^. The $f\left(\alpha_{0}\right)$ and $\;f\left(\alpha_{c}\right)$ values can be determined by Equations (5)–(8):

(5) $$f\left(\alpha_{0}\right)=\frac{1.99-\alpha_{0}(1-\alpha_{0})(2.15-3.93\alpha_{0}-2.7{\alpha_{0}}^{2})}{\left(1+2\alpha_{0}\right){\left(1-\alpha_{0}\right)}^{\frac{3}{2}}},\alpha_{0}=\frac{a_{0}}{h},$$

(6) $$f\left(\alpha_{c}\right)=\frac{1.99-\alpha_{c}(1-\alpha_{c})(2.15-3.93\alpha_{c}-2.7{\alpha_{c}}^{2})}{(1+2\alpha_{c})(1-\alpha_{c}{)}^{\frac{3}{2}}},\alpha_{c}=\frac{a_{c}}{h},$$

(7) $$a_{c}=\frac{2}{\pi }\left(h+h_{0}\right)\arctan{(\frac{tE}{32.6P_{\max}}{\mathrm{CMOD}}_{c}-0.1135)}^{\frac{1}{2}},$$

(8) $$E=\frac{1}{tc_{i}}\left[3.70+32.60\tan^{2}(\frac{\pi }{2}\frac{a_{0}+h_{0}}{h+h_{0}})\right],$$

where CMOD_c_ is the critical crack opening displacement and $c_{i}$ denotes the initial slope of the *P*-CMOD curves.

**Figure 24:**
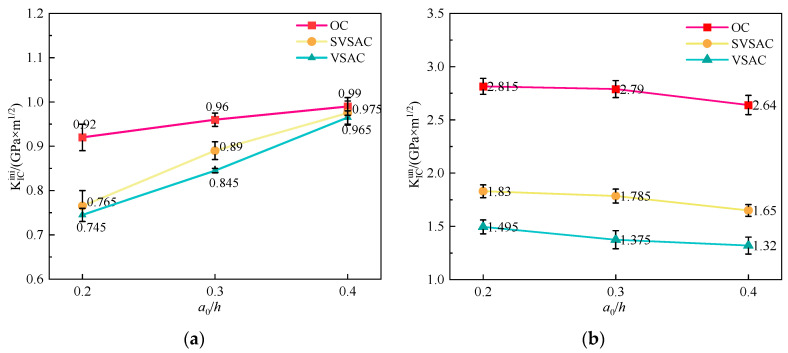
The variation in fracture toughness: (**a**) $K_{\mathrm{IC}}^{\mathrm{ini}}$; (**b**) $K_{\mathrm{IC}}^{\mathrm{un}}$.

2. Fracture toughness.

(1) Effect of mix components;

The fracture toughness of SVSAC was affected by concrete mix components (Table 4). The coarse aggregate significantly changed the $K_{\mathrm{IC}}^{\mathrm{ini}}$ and $K_{\mathrm{IC}}^{\mathrm{un}}$ values of concrete (Figure 24a,b). In general, the fracture toughness of concrete with VSCA was lower than that of concrete with gravel. Compared to OC, the $K_{\mathrm{IC}}^{\mathrm{ini}}$ values of VSAC and SVSAC decreased by 11.18% and 8.55%, respectively (Table 4), and the $K_{\mathrm{IC}}^{\mathrm{un}}$ values of VSAC and SVSAC were 49.20% and 36.17% lower, respectively. 

However, the fracture toughness of the SVSAC specimens was higher than that of the VSAC specimens owing to the effects of SW and SS. Compared with VSAC, the $K_{\mathrm{IC}}^{\mathrm{ini}}$ and $K_{\mathrm{IC}}^{\mathrm{un}}$ of SVSAC increased by 3.03% and 25.74%, respectively. The high concentration of Cl^−^ ions in SW and SS can improve the microstructure of the concrete. Additionally, the formation of salts, such as Friedel’s salt, can fill the voids in very small capillary pores, enhancing the fracture toughness of the concrete.

(2) Effect of *a*_0_/*h*.

The effect of *a*_0_/*h* on fracture toughness is negligible [49]. The test results indicated that the $K_{\mathrm{IC}}^{\mathrm{ini}}$ and $K_{\mathrm{IC}}^{\mathrm{un}}$ values of SVSAC were approximately 0.88 and 1.79, respectively (Figure 24a,b).

#### 3.6.2. *σ*-CTOD Relations

The *σ*-CTOD relation (tension-softening curve) represents the relation between the tensile stress *σ* and CTOD in the FPZ of concrete, which reveals the ability of the concrete cracking to transmit *σ*.

**Figure 25:**
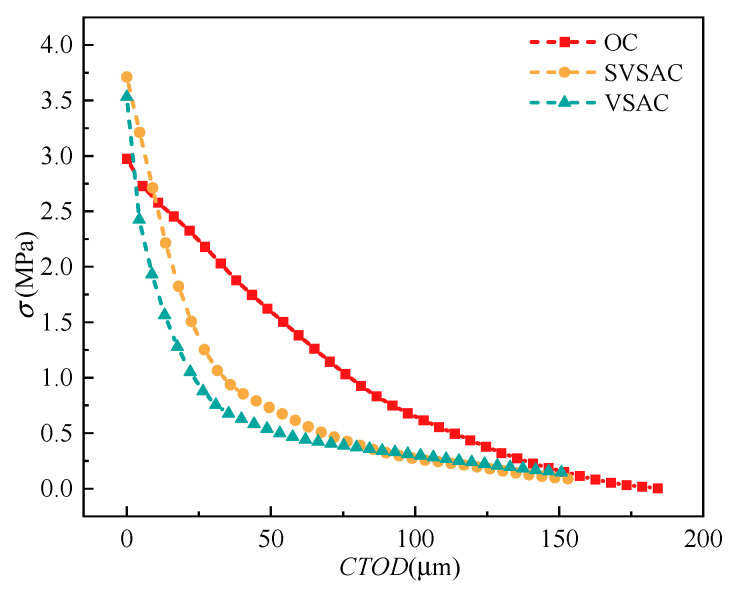
*σ*-CTOD curves of concrete.

In general, it was difficult to directly obtain *σ*-CTOD relations through the three-point bending beam test due to the non-uniform distribution of the cohesive force and the COD [50]. Thus, the J-integral method combined with the *P*-CTOD curve was used to derive the tensile-softening behavior of the concrete [51]. According to the analyses and results, the *σ*-CTOD relations of SVSAC are shown in Figure 25. 

Compared with OC, the *σ*-CTOD curves of specimens adopting VSCA declined more suddenly. This indicated that *σ* transmitted by the cracking surface of SVSAC was significantly inferior to OC under a similar crack width condition. It was also found that the *σ* values of OC at CTOD = 20, 50, and 100 μm were 2.37, 1.59, and 0.65 MPa, respectively; however, those of SVSAC were 1.66, 0.72, and 0.27 MPa, respectively. This can be attributed to the inferior mechanical properties of VSCA (porosity and low strength) and destruction of the coarse aggregate.

Furthermore, the *σ*-CTOD of SVSAC dropped relatively slowly in comparison with VSAC. The *σ* values of SVSAC at CTOD = 20 and 50 μm were 44.35% and 38.46% greater than those of VSAC at the same CTOD. SW and SS could efficiently improve the capacity of SVSAC cracking to bear and transmit *σ*. Finally, the effects of SW and SS on the *σ*-CTOD relation became negligible when CTOD reached 100 μm (0.1 mm), and the *σ* values of SVSAC and VSAC were 0.27 and 0.30 MPa, respectively. The coarse aggregate affects the entire crack propagation process; however, SS and SW have a notable impact on the early stages of crack propagation, and their effect becomes minor when the CTOD reaches 0.1 mm.

## 4. Theoretical Analyses of Tension-Softening Curve

### 4.1. General Expression

The addition of SW, SS, and VSCA could cause changes in the shape of the *σ*-CTOD curve of SVSAC based on the test results, which indicates a variation in the mechanism of fracture. Thus, theoretical models of tension-softening relationships considering the coupled effects of concrete mix components need to be established to describe these variations. Based on the experimental results and Ref. [52], the analytical expression of the *σ*-CTOD relations of SVSAC was obtained through Matlab (Matlab 24.1.0.2508561 (R2024a))analysis, as shown in Equation (9).

(9) $$\frac{\sigma }{f_{\kappa }}=[1+{\left(a\frac{\mathrm{CTOD}}{{\mathrm{CTOD}}_{0}}\right)}^{3}]e^{-\frac{\mathrm{CTOD}}{{\mathrm{CTOD}}_{0}}}-\frac{\mathrm{CTOD}}{{\mathrm{CTOD}}_{0}}(1+a^{3})e^{-b}$$

### 4.2. Critical Parameters

The factors in Equation (9), *a* and *b*, significantly affect the characteristics of the tension-softening curve. The *a* and *b* in Equation (9) are critical parameters considering the effects of the mix components, and they can be derived through the analysis of the experimental data using a numerical regression analysis program coded in Matlab software (Equations (10) and (11)). 

Furthermore, the suggested equations were applicable to the specimen with 8.49% ≤ *k* ≤ 26.4%, 0% ≤ *γ*_1_ ≤ 1.983%, and 0% ≤ *γ*_2_ ≤ 0.1%.

(10) $$a=0.1899k-0.9382\gamma_{1}-4.1627\gamma_{2}-0.4468,$$

(11) $$b=0.5473k-1.9298\gamma_{1}-6.2166\gamma_{2}-3.5812,$$

**Figure 26:**
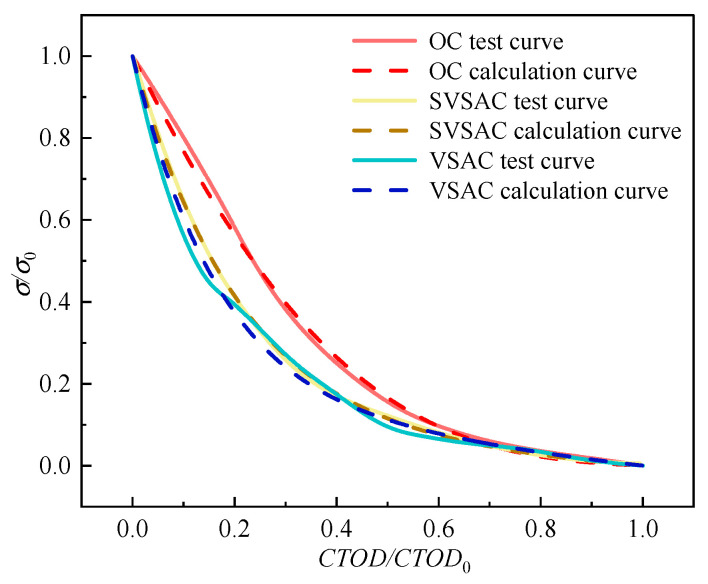
Comparison of softening curves between test and calculation.

A comparison between the experimental and calculated curves is shown in Figure 26. The differences were acceptable, and the ability of the SVSAC cracking to transmit *σ* was reasonably predicted by the analytical model. The obtained curves are in good agreement with the real curves.

## 5. Numerical Simulation Analyses of Fracture

Nonlinear finite element (FE) analyses of SVSAC fracture were undertaken by ABAQUS (Abaqus 6.14) software, and changes in the fracture mechanism and macro-mechanical properties of SVSAC under various conditions were systematically studied.

### 5.1. FE Model of SVSAC Specimen

#### 5.1.1. FE Types and Material Properties 

The FE element of SVSAC adopted a general-purpose linear solid brick element C3D8R with reduced integration, which could properly describe the plastic properties and damage evolution of materials. The advantage of using C3D8R for concrete modeling is time-saving and an acceptable results’ accuracy. 

Furthermore, the element of the loading steel plate of the three-point bending beam was C3D8.

In general, ABAQUS software adopted three different constitutive models to describe the mechanical properties and mechanism of concrete, i.e., the smeared crack model, damaged plasticity model, and brittle cracking model. Based on the fracture test, the brittle cracking model was used in this study owing to it being the most accurate in applications where brittle behavior dominates. Thus, this model was combined with the suggested tension-softening relation of SVSAC (Equation (9)) to investigate the variations in the fracture mechanism and macro-properties of specimens under different conditions. 

The elasto-plastic constitutive model was proposed for the steel plate, and the related stress–strain curve refers to Ref. [53].

#### 5.1.2. Establishment of FE Model of Fracture Specimen

**Figure 27:**
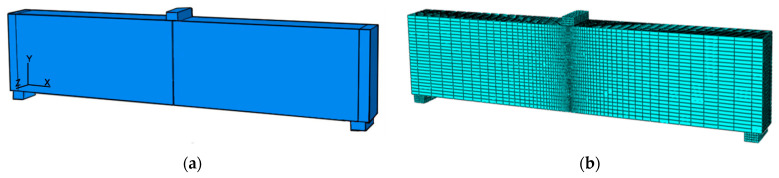
FE model of specimen: (**a**) Geometric entities of specimen; (**b**) FE mesh of specimen.

The establishment of the nonlinear FE model of the SVSAC three-point bending specimen mainly consisted of two steps. First, the geometric entities of the beam and loading plate were built (Figure 27a). Second, the element type, material properties, and constitutive model were assigned, and the entities were meshed (Figure 27b). Third, the boundary of the beam (two ends) was fixed and the displacement loading program was adopted according to the actual test. Finally, numerical simulations of the specimen were performed under different conditions.

It should be noted that the proper size of elements was determined after the preliminary studies. The element size of concrete at the tip of beam notch kept 0.5 mm (height) × 3 mm (length) × 3 mm (width), while the size of the other elements was approximately 3–30 mm.

#### 5.1.3. Validity of FE Model

**Figure 28:**
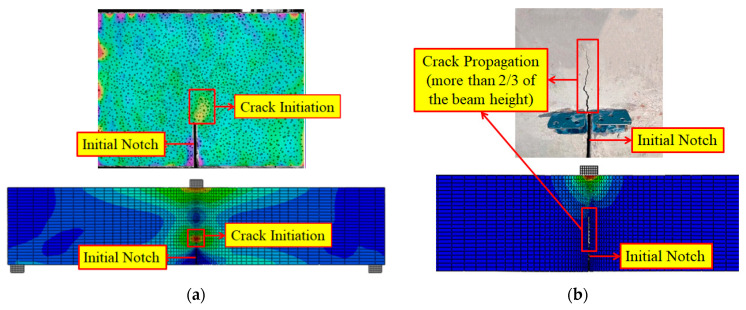
Comparison between SVSAC numerical results and test results: (**a**) Pre-80%; (**b**) Post-40%.

**Figure 29:**
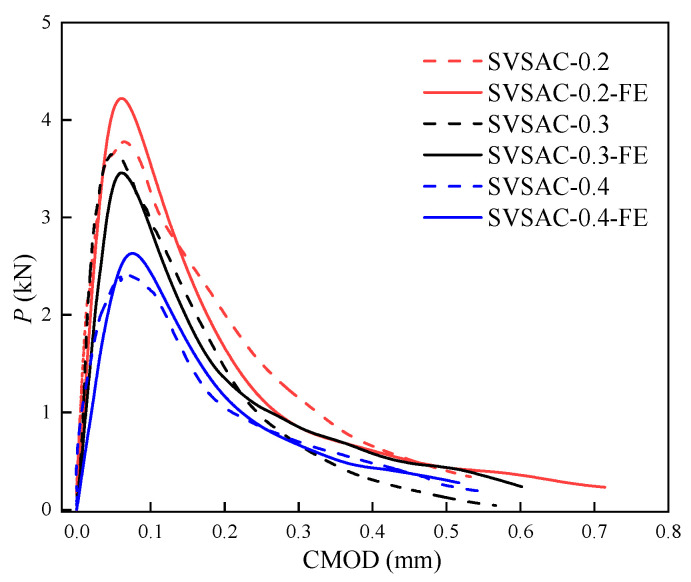
*P*-CMOD curves of SVSAC from experiment and simulation.

A comparison between the numerical results and test results is shown in Figure 28 and Figure 29. It was obtained that the model predicted the fracture response of SVSAC with acceptable accuracy. The simulated failure process and *P*-CMOD curves of SVSAC were in line with the test data. Thus, the suggested numerical model and *σ*-CTOD relation (Equation (9)) were adopted to study the fracture mechanism and macro-mechanical properties of SVSAC under critical variables (e.g., sizes of specimen).

### 5.2. Numerical Results of SVSAC

Generally, several critical fracture parameters of concrete greatly changed with variation in the size of the specimen [54,55], particular its fracture toughness ($K_{\mathrm{IC}}^{\mathrm{ini}}$ and $K_{\mathrm{IC}}^{\mathrm{un}}$) and *P*-CMOD curve. However, it is difficult to obtain the independent effect on the fracture of concrete experimentally. Thus, numerical simulations were undertaken and the changes in fracture properties was obtained.

**Figure 30:**
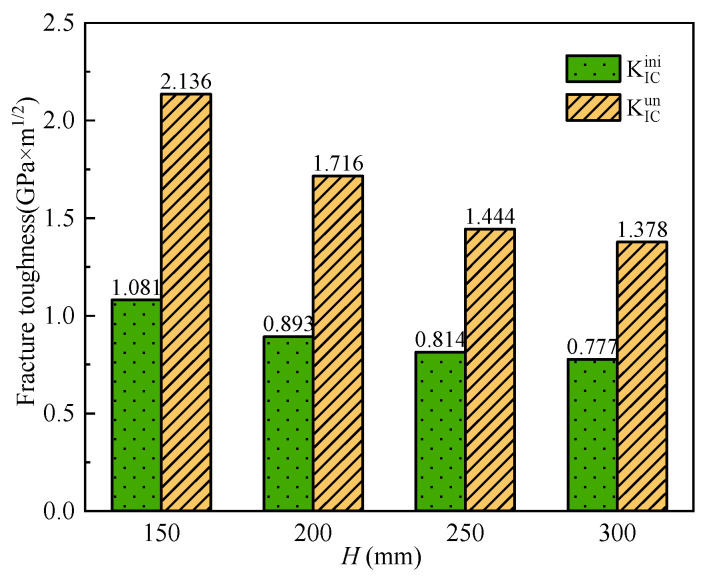
Variation in fracture toughness.

Four different heights (*H* = 150, 200, 250, and 300 mm), lengths (*L* = 900, 1190, 1500, and 1800 mm), and widths (*W* = 80, 100, 120, and 140 mm) of the SVSAC beam were considered in the FE analyses, and the variations in *P*_max_, $K_{\mathrm{IC}}^{\mathrm{ini}}$, and $K_{\mathrm{IC}}^{\mathrm{un}}$ are listed in Table 5. It was observed that *P*_max_ increased with increasing *H* and *W* while decreasing *L*. The higher (lower) the *H* and *W* (*L*), the greater the stiffness of the specimen, and the greater the *P*_max_. Furthermore, the fracture toughness ($K_{\mathrm{IC}}^{\mathrm{ini}}$ and $K_{\mathrm{IC}}^{\mathrm{un}}$) was obviously improved through increasing *L* and decreasing *H* (Figure 30). The test results indicated that the $K_{\mathrm{IC}}^{\mathrm{ini}}$ and $K_{\mathrm{IC}}^{\mathrm{un}}$ of specimens with 300 mm *H* were 28% and 35% inferior compared with those of specimens with 150 mm *H*, respectively. This phenomenon can be attributed to the toughness of the beam being significantly reduced by an improvement in structure stiffness [56].

**Figure 31:**
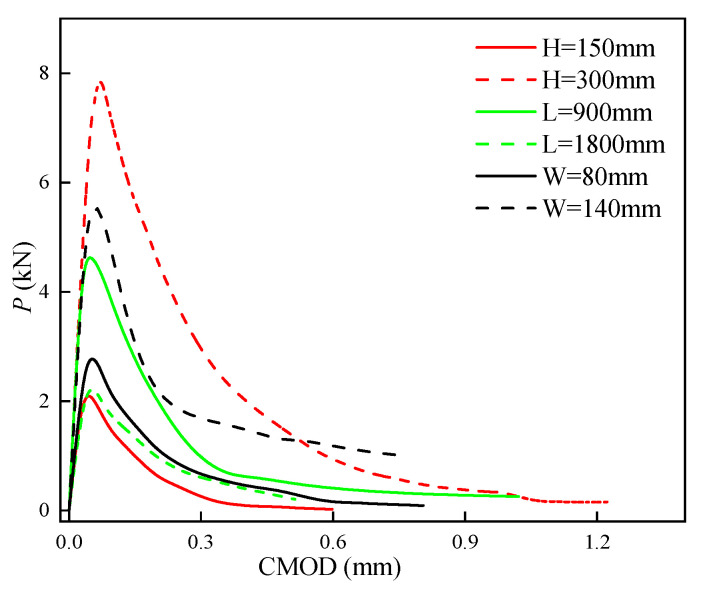
SVSAC P-CMOD curves under different size conditions.

The characteristics of the *P*-CMOD curves of SVSAC also changed with a variation in the size of the specimen (Figure 31). It was obtained that the descending portion of the curve became steep after increasing *H* and decreasing *L*, which caused inferior deformability and unstable fracture toughness ($K_{\mathrm{IC}}^{\mathrm{un}}$) of SVSAC. Furthermore, the critical CMOD (CMOD_c_) was also improved through enhancing the size of the specimen. Generally, the CMOD_c_ of specimens with 300 mm *H* was 35.9% greater compared with that of specimens with 150 mm *H*.

### 5.3. Analytical Model of Fracture Toughness Considering Size Effect and Mix Component

Based on the simulation results and the experimental data, the fracture toughness ($K_{\mathrm{IC}}^{\mathrm{ini}}$ and $K_{\mathrm{IC}}^{\mathrm{un}}$) of SVSAC was significantly affected by the concrete mix components and specimen size. However, few studies have focused on this field, and few analytical models have been used to characterize the changes in fracture toughness caused by mix components and size components. 

Thus, according to Refs. [32,57,58,59,60,61] and the results, a theoretical model was developed to consider the effects of SW, SS, VSCA, and specimen size. Matlab software was also used to code a numerical program for the regression analysis, which was adopted to investigate the experimental and numerical data and obtain a specific formula. The analytical expressions for $K_{\mathrm{IC}}^{\mathrm{ini}}$ and $K_{\mathrm{IC}}^{\mathrm{un}}$ are expressed as Equations (12) and (13):

(12) $$K_{\mathrm{IC}}^{\mathrm{ini}}=CR\left(0.0837+21.4751CR^{-2}\right)+0.0799\gamma_{1}+0.08\gamma_{2}-5.247(\frac{H}{L}{)}^{2}+3.337\frac{H}{L}-2.762,$$

(13) $$K_{\mathrm{IC}}^{\mathrm{un}}=1.5897{\left(CR-7.5094\right)}^{-1.5272}+0.2294\gamma_{1}+2.18\gamma_{2}+14.687{\left(\frac{H}{L}\right)}^{2}-8.031\frac{H}{L}+2.0362$$

where *γ*_1_ and *γ*_2_ are the Cl^−^ content of SW and SS, respectively, *CR* is the crushing index of the coarse aggregate, and *H* and *L* are the height and length of the beam, respectively. To verify the application and reliability of the theoretical model, the calculated results and experimental data obtained in Refs. [27,52,53,54,55,56] are also listed in Table 6.

The calculated fracture toughness was consistent with the actual values, and the standard deviations of $K_{\mathrm{IC}}^{\mathrm{ini}}$ and $K_{\mathrm{IC}}^{\mathrm{un}}$ were just 0.018 MPa·m^1/2^ and 0.036 MPa·m^1/2^, respectively. The difference was acceptable, and the theoretical model can accurately describe the fracture properties of SVSAC. Furthermore, the suggested models were applicable to the specimen with 8.49% ≤ *k* ≤ 26.4% and *γ*_1,2_ ≤ 2.083%. However, there were few studies focused on the fracture of SVSAC. Further research works need to be undertaken to improve the accuracy of this theoretical model.

## 6. Conclusions 

The typical fracture properties of SVSAC were investigated through experimental and theoretical analyses. The main conclusions are as follows.

(1) The failure characteristics of three-point bending beams were significantly changed after adopting VSCA. Compared to the OC specimen, the crack initiation of SVSAC was earlier (60% of *P*_max_), and the cracking width and length of the SVSAC specimen increased by 4.88% and 28.32%, respectively. The adoption of VSCA accelerated the crack initiation and propagation of concrete, while SW and SS could inhabit its influence before 80% of *P*_max_ (post peak point).
(2) The fracture characteristic loads (i.e., *P*_ini_ and *P*_max_), *G*_f_, and *L*_ch_ of the SVSAC specimen decreased with increasing *a*_0_/*h*, and VSCA enhanced these effects. The *P*_ini_ (*P*_max_) of specimens adopting VSCA was 10.94% (9.47%) inferior to that of the OC specimen, and this difference became small (4%) after using SW and SS. The fracture energies of the SVSAC and VSAC specimens were significantly decreased. Generally, the *L*_ch_ value of SVSAC was, on average, 59.49% lower than that of OC.
(3) The *P*-CTOD and *σ*-CTOD curves of SVSAC were characterized by rapid decline. The effect of *a*_0_/*h* on fracture toughness was minor, while the $K_{\mathrm{IC}}^{\mathrm{ini}}$ and $K_{\mathrm{IC}}^{\mathrm{un}}$ of SVSAC decreased by 8.55% and 36.17% in comparison with those of OC, respectively. The tensile stress transmitted by the cracking surface of specimens adopting VSCA was significantly inferior to OC under a similar crack width, and this difference can be partly improved through using SW and SS in the early stage of loading. The improvements of SW and SS on the cracking and *σ* transmission of SVSAC became negligible when CTOD reached 0.1 mm.
(4) A finite element model was developed to analyze the fracture behavior of SVSAC. The numerical results showed that the fracture toughness ($K_{\mathrm{IC}}^{\mathrm{ini}}$ and $K_{\mathrm{IC}}^{\mathrm{un}}$) was greatly improved through changing the size of the specimen, i.e., beam height, length, and width. The *P*-CMOD curves of SVSAC became steep after increasing the specimen height and width.
(5) Analytical models for the double-*K* fracture toughness and *σ*-CTOD relation of SVSAC considering the effects of mix components and specimen size were established. The fracture properties of SVSAC would be efficiently predicted by the models.
(6) The VSCA, SW, and SS could change the micro- and macrostructures of concrete, causing variations in the fracture of SVSAC. Thus, further research works should be performed together with more testing techniques (e.g., XRF, XRD, X-ray spectroscopy) to deeply and completely investigate the performance of SVSAC.

## Figures and Tables

**Table 1:** Properties of the coarse and fine aggregates.

Materials	Size (mm)	*ρ*_b_ (kg·m^−3^)	*ρ*_a_ (kg·m^−3^)	*ω*_a_ (kg·m^−3^)	*CL* (%)	*CR* (%)	*α* (%)
Gravel	5.0–31.5	1560	2590	1.2	-	8.49	-
VSCA	5.0–31.5	708	1647	14.81	-	26.4	-
SS	0.15–4.75	1579	-	-	0.72	-	1.8
RS	0.15–4.75	1611	-	-	2.90	-	-

Note: *ρ*_b_, *ρ*_a_, *ω*_a_, *CL*, *CR*, and *α* denote the bulk density, the apparent density, water absorption, clay content, crushing index, and shell content of the aggregates, respectively.

**Table 2:** Mix proportion of concrete (kg·m^−3^).

Concrete Type	Cement	FW	SW	RS	SS	Gravel	VSCA	Water-Reducing Agent
OC	285	190	-	676	-	1202	-	-
SVSAC	459	-	265	-	834	-	638	0.4
VSAC	459	256	-	834	-	-	638	0.4

**Table 3:** Parameters of the specimen.

Specimen Number	Coarse Aggregates	Fine Aggregates	Water	*a*_0_/*h*
OC-0.2	Gravel	RS	FW	0.2
OC-0.3	Gravel	RS	FW	0.3
OC-0.4	Gravel	RS	FW	0.4
SVSAC-0.2	VSCA	SS	SW	0.2
SVSAC-0.3	VSCA	SS	SW	0.3
SVSAC-0.4	VSCA	SS	SW	0.4
VSAC-0.2	VSCA	RS	FW	0.2
VSAC-0.3	VSCA	RS	FW	0.3
VSAC-0.4	VSCA	RS	FW	0.4

**Table 4:** Physical properties of concrete and fracture parameters.

Specimen Number	Concrete Type	*f*_cu_ (MPa)	*f*_c_ (MPa)	*f*_t_ (MPa)	*E*_c_ (GPa)	*ρ*_d_ (kg·m^−3^)	*P*_ini_ (kN)	*P*_max_ (kN)	$K_{Ic}^{ini}$ (MPa·m^1/2^)	$K_{Ic}^{un}$ (MPa·m^1/2^)	*a_c_* (mm)	*G_f_* (N·m^−1^)
OC-0.2	OC	38.3	33.2	2.83	24.2	2208	2.73	4.77	0.92	2.815	0.097	154.955
OC-0.3							2.16	3.72	0.96	2.79	0.1165	132.05
OC-0.4							1.62	2.86	0.99	2.64	0.127	117.29
SVSAC-0.2	SVSAC	39.2	37.2	3.22	17.1	1836	2.66	4.31	0.765	1.83	0.0865	115.8
SVSAC-0.3							1.94	3.50	0.89	1.785	0.0895	83.45
SVSAC-0.4							1.40	2.62	0.975	1.65	0.099	73.3
VSAC-0.2	VSAC	37.5	36.6	3.14	16.6	1809	2.53	3.95	0.745	1.495	0.076	102.7
VSAC-0.3							1.87	3.13	0.845	1.375	0.079	78.8
VSAC-0.4							1.32	2.19	0.965	1.32	0.0915	72.1

**Table 5:** Fracture behaviors of SVSAC under different size conditions.

Specimen Number	*H* (mm)	*L* (mm)	*W* (mm)	*P*_max_ (kN)	$K_{Ic}^{ini}$ (MPa·m^1/2^)	$K_{Ic}^{un}$ (MPa·m^1/2^)
1	150	1190	100	2.093	1.081	2.136
2	200	1190	100	3.477	0.893	1.716
3	250	1190	100	5.166	0.814	1.444
4	300	1190	100	7.834	0.777	1.378
5	200	900	100	4.627	0.754	1.146
6	200	1190	100	3.477	0.893	1.716
7	200	1500	100	2.695	1.188	2.433
8	200	1800	100	2.206	1.528	3.351
9	200	1190	80	2.771	0.929	1.714
10	200	1190	100	3.477	0.893	1.716
11	200	1190	120	4.161	0.919	1.712
12	200	1190	140	5.544	0.986	1.741

**Table 6:** Measurement and calculation of fracture toughness.

Specimen	*γ* (g/L)	*k* (%)	$K_{IC}^{ini}$ Test Value (MPa·m^1/2^)	$K_{IC}^{ini}$ Calculated Value (MPa·m^1/2^)	$K_{IC}^{un}$ Test Value (MPa·m^1/2^)	$K_{IC}^{un}$ Calculated Value (MPa·m^1/2^)
OC	0	8.49	0.957	0.927	2.748	2.739
VSAC	0	26.4	0.852	0.711	1.397	1.119
SVSAC	2.08	26.4	0.877	0.880	1.755	1.660
HHL-0.4 [32]	1.84	9.605	0.980	0.923	2.000	2.022
C40-1 [57]	0	/	0.847	0.849	1.504	1.598
SE30-1 [58]	0	/	0.802	0.845	1.560	1.550
0.3-1 [59]	0	/	1.034	1.034	2.072	2.073
MG100 [60]	0	26.2	0.680	0.718	0.890	9.048
RAC100 [61]	0	13	0.472	0.472	1.143	1.135

## Data Availability

Data are contained within the article.
